# Bioactivity of extracts from *Gentiana dinarica* (Beck.) plants collected in natural habitat and cultured in vitro

**DOI:** 10.1038/s41598-026-58820-4

**Published:** 2026-06-18

**Authors:** Sara Milijanovic, Jelena Kulas, Dusanka Popovic, Dijana Krstic-Milosevic, Gordana Tovilovic-Kovacevic, Dijana Bovan, Teodora Komazec, Svetlana Sokovic Bajic, Ivana Mirkov, Dina Tucovic

**Affiliations:** 1 Immunotoxicology group, Department of Ecology, Institute for Biological Research “Sinisa Stankovic”–National Institute of the Republic of Serbia, University of Belgrade, Bulevar despota Stefana 142, Belgrade, 11000 Serbia; 2 Department of Plant Physiology, Institute for Biological Research “Sinisa Stankovic” – National Institute of the Republic of Serbia, University of Belgrade, Belgrade, Serbia; 3 Department of Biochemistry, Institute for Biological Research “Sinisa Stankovic” – National Institute of the Republic of Serbia, University of Belgrade, Belgrade, Serbia; 4 Department of Immunology, Institute for Biological Research “Sinisa Stankovic” – National Institute of the Republic of Serbia, University of Belgrade, Belgrade, Serbia; 5 Group for Probiotics and Microbiota-Host Interaction, Department for Microbiology and plant biology, Institute of Molecular Genetics and Genetic Engineering, University of Belgrade, Belgrade, Serbia

**Keywords:** *Gentiana dinarica*, Plants cultured in vitro, Antioxidant capacity, Antibacterial activity, Antitumor activity, Immunomodulation, Biochemistry, Biological techniques, Biotechnology, Microbiology, Plant sciences

## Abstract

Differences in the chemical composition of extracts obtained from plants grown in natural habitats and those of the same species grown in plant tissue culture may lead to variations in extract activities. Antioxidant, antibacterial, antitumor, and immunomodulatory activities were compared for extracts from the aerial parts and roots of *G. dinarica* (derived from natural habitats or cultured in vitro). An increase in secoiridoid and xanthone contents in extracts from shoots cultured in vitro, compared to aerial parts of plants from natural habitats, resulted in improved antibacterial activity against *Pseudomonas aeruginosa* (but reduced activity against *Staphylococcus aureus*), and better selectivity and activity against tumor cell lines. An anti-inflammatory response (decreased myeloperoxidase (MPO), interleukin (IL)-6, IL-17, and interferon (IFN)-γ production) was observed in spleen cells following stimulation with extract from aerial parts, whereas stimulation with shoot culture extract induced a pro-inflammatory response (increased IL-6, IL-17, and IFN-γ production). Roots cultured in vitro lack secoiridoids, which are present in roots of plants from natural habitats, and accumulate higher amounts of xanthones, resulting in differences in certain activities. Production of immune mediators was altered only in response to extract from roots of plants from a natural habitat (increased IL-6 and nitric oxide (NO)), while extracts from roots cultured in vitro exhibited the strongest free radical scavenging capacity and showed better activity against *P. aeruginosa*. Depending on the activity tested, potentiation, suppression, or absence of effect may be observed, indicating the need for a detailed comparison of the biological activities of extracts obtained from plants grown in natural habitats and tissue cultures that differ in chemical composition.

## Introduction

*Gentiana dinarica* (Gentianaceae) is an endemic and endangered plant species found in the Dinaric Alps and Apennines^1^. Like other *Gentiana* species, it has been used in traditional medicine to stimulate appetite, relieve stomach aches^2,3^, prevent haemorrhages^3^, and treat migraines^2^. As this species is rare and threatened, its wider use is limited. However, plant protection and preservation through biotechnology based on plant tissue culture methods could conserve it and enable large-scale biomass production with stable chemical content in the plant material. For *G. dinarica*, a protocol for in vitro cultivation of shoots and adventitious roots was established by Vinterhalter et al.^4^.

Although it is generally accepted that more stable chemical content in plant material can be achieved during in vitro propagation by eliminating environmental variations, quantitative and/or qualitative differences in the chemical composition of wild plants and those propagated in vitro are common and well documented^5–7^. The qualitative composition of secondary metabolites in *G. dinarica* grown in vitro and collected from its natural habitats shows no differences; however, quantitative analysis indicates that shoot cultures contain higher amounts of secoiridoids and xanthones than plants from the natural habitats^4^. Roots propagated in vitro lack secoiridoids, which are present in roots from the natural habitat, and also contain higher levels of xanthones^4^.

Due to quantitative and/or qualitative differences in chemical composition, differences in the activity, mainly antioxidant and antibacterial, of extracts from plants grown in natural habitats and those cultured in vitro have been observed^5–8^. For example, due to higher phenol content in the extracts, stronger antibacterial and antioxidant activity was observed in extracts of *Ephedra* sp. from natural habitat compared to those cultured in vitro^7^. In contrast, extracts obtained from *Verbena officinalis* plants grown in vitro were more effective than those from soil-grown plants^8^. For *G. dinarica*, extracts from roots grown in vitro showed stronger inhibition of α-glucosidase compared to extracts from roots collected from natural habitats, whereas no differences were noted between extracts of aerial parts from plants grown in natural habitats and shoots cultured in vitro^9^. Additionally, a more pronounced antiglioma effect (a greater decrease in viability of the U251 cell line) was observed in extracts from roots cultured in vitro compared to roots from natural habitats^10^. The observed differences in the activity of *G. dinarica* extracts have been associated with the higher xanthone content in the cultured roots^9,10^. To our knowledge, apart from these two studies and our previous work examining the immunomodulatory potential of *G. dinarica* aerial parts from natural habitats^11^, there are no other data regarding the activity of extracts from this plant.

In this context, the present study aimed to determine whether and how the secondary metabolite composition of plant extracts derived from *G. dinarica* grown in natural habitats (aerial parts and roots) and propagated in vitro (shoot and root cultures) influences their antioxidant, antimicrobial, antitumor, and immunomodulatory activities.

## Materials and methods

### Plant material and extract preparation

**Fig. 1:**
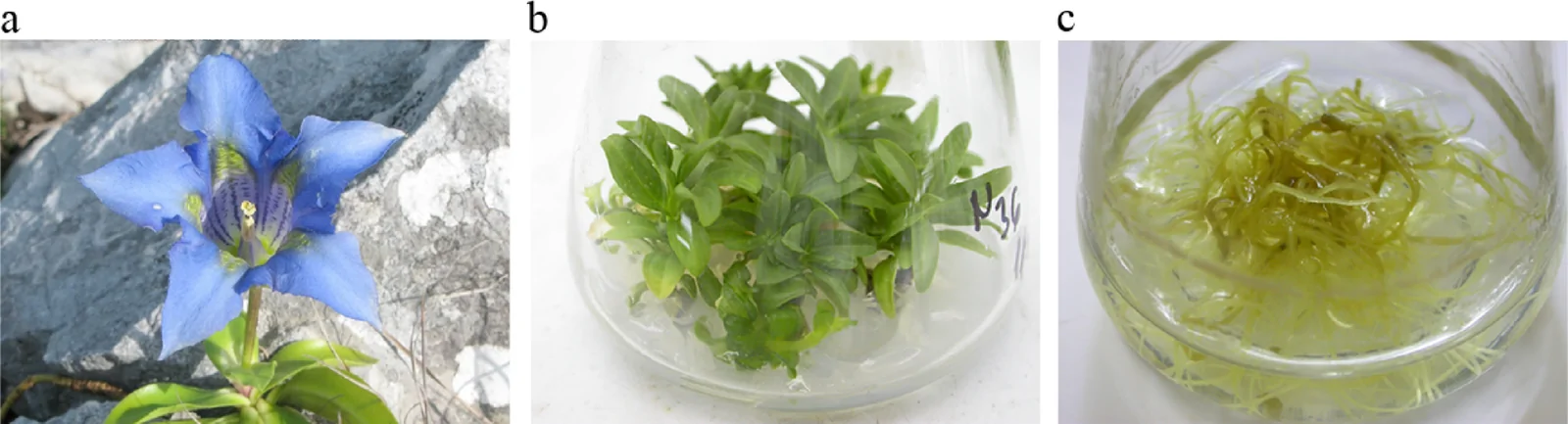
*G. dinarica* in its natural habitat and cultured in vitro. (**a**) Plant in its natural habitat on Tara Mountain (approximately 1,300 m above sea level), western Serbia. Shoots cultured in vitro (**b**) and roots cultured in vitro (**c**) after 5 weeks of cultivation.

The plants of *G. dinarica* (Fig. 1a) used as starting material for extract preparation were collected in their native habitats at the flowering stage and identified by Snezana Jaric (Department of Ecology, Institute for Biological Research “Sinisa Stankovic” – National Institute of the Republic of Serbia, University of Belgrade, Belgrade, Serbia). Permission for collecting plant material was obtained from the Ministry of Environmental Protection (353-01-644/2021-04, dated 19th April 2021). The collection of plant material complies with the IUCN Policy Statement on Research Involving Species at Risk of Extinction and the Convention on the Trade in Endangered Species of Wild Fauna and Flora. Herbarium specimens of the studied species have been deposited in the herbarium of the Institute for Biological Research ‘Sinisa Stankovic’—National Institute of the Republic of Serbia (IBISS) in Belgrade. *G. dinarica* Beck (voucher specimen Gen-G-din-03/21-IBISS) was collected on Mount Tara (location: Drlije, NP ‘Tara’).

The propagation of *G. dinarica* followed a standard micropropagation technique, using axillary buds from plants collected in their natural habitat as starting material. A detailed propagation protocol was published in Vinterhalter et al.^4^. In brief, *G. dinarica* was maintained as shoot cultures (Fig. 1b) on a basal medium containing MS^12^ mineral salts, LS^13^ vitamins, 100 mg/L myo-inositol, 2% (w/v) sucrose, solidified with 0.64% (w/v) agar, and supplemented with 1.0 mg/L 6-benzyladenine and 0.1 mg/L α-naphthaleneacetic acid. Excised root cultures (Fig. 1c), established from the tips of adventitious roots that developed in shoot cultures, were grown on basal half-strength MS liquid medium without agar, with 0.5 mg/L indole-3-butyric acid, on an orbital shaker at 85 rpm. Cultures were maintained at 25 ± 2 °C in a controlled environment room illuminated with cool-white fluorescent tubes providing a flux rate of 40 µmol/s/m^2^ for shoots and 2 µmol/s/m^2^ for roots under a 16-hour (long day) photoperiod and were subcultured onto fresh medium every 5 weeks.

Methanol extracts were prepared from the aerial parts and roots of *G. dinarica* collected in the natural habitat, as well as from shoots and roots cultured in vitro. Air-dried plant material was ground using an electric mill and extracted with methanol in an ultrasonic bath for 20 min. The ratio of plant material to solvent was 1:10, w/v. After sonication, extraction continued by maceration with occasional shaking in the dark at room temperature for 24 h. The extracts were then filtered and evaporated to dryness using a vacuum rotary evaporator (Buchi R-210, Flawil, Switzerland) at 50 °C. The extraction yields were 28.3% and 24.8% for the aerial parts and roots of plants collected in the natural habitat, respectively. For shoots and roots obtained in vitro, the extraction yields were 20.7% and 16.9%, respectively. The extracts were stored at 4 °C until further analysis.

### HPLC analysis

Secondary metabolites, including xanthones and secoiridoids, in *G. dinarica* methanol extracts were determined using the HPLC method previously published by Krstić-Milošević et al.^14^. The analysis was performed on an Agilent 1100 series HPLC instrument (Agilent, Waldbronn, Germany), with a DAD detector, using a reverse phase Zorbax SB-C18 (Agilent) analytical column (150 mm × 4.6 mm i.d., 5 μm particle size) and a degassed mobile phase consisting of two solvents: 0.1% v/v orthophosphoric acid in water (A) and acetonitrile (B) at a flow rate of 1 mL/min. Before HPLC analysis, the extracts were filtered through nylon syringe filters (Captiva syringe filters, 0.45 μm, 13 mm, Agilent Technologies). The gradient elution was as follows: 98–90% A 0–5 min, 90% A 5–10 min, 90–85% A 10–13 min, 85% A 13–15 min, 85–70% A 15–20 min, 70–40% A 20–24 min, 40–0% A 24–28 min. Detection wavelengths were set at 260 and 320 nm. The injection volume of samples was 5 µL. Commercial standards of the secoiridoids swertiamarin, gentiopicrin, and sweroside were purchased from Cfm Oscar Tropitzsch (Bayern, Germany). The isolation, identification, and characterization of the xanthones norswertianin-1-*O*-primeveroside, gentioside, norswertianin, and *C*-glucoflavone isoorientin were reported in previous studies^15,16^. Identification of secondary metabolites was confirmed by the co-injection method, using standard compounds. Quantification was performed using calibration curves in an external standard method. Stock solutions of standards were prepared by dissolving the compounds in methanol to obtain five different concentrations for calibration curves. Analyses were performed at least twice. The results are presented as mg per g of dry extract.

### Antioxidant capacity of extracts

The antioxidant capacity of extracts from wild- and in vitro-grown plants was evaluated using DPPH (2,2-diphenyl-1-picrylhydrazyl) and ABTS (2,2′-azino-bis-3-ethylbenzotiazolin-6-sulfonic acid) free radical assays, with slight modifications^17,18^. In brief, various concentrations of investigated methanol extracts (12.5–200 µg/mL) were mixed with DPPH methanol solution (final concentration 150 µM) in a 1:1 ratio and incubated for 30 min at room temperature in the dark. The absorbance of the remaining DPPH was measured spectrophotometrically at 517 nm using an automated microplate reader (Multiskan Spectrum; Thermo Fisher Scientific, Waltham, MA, USA). Ascorbic acid was used as the standard antioxidant compound. The results were expressed as % of residual DPPH radical, with the DPPH solution without extract/ascorbic acid set as 100%.

The ABTS test was performed by generating ABTS radical cations and exposing them to the investigated plant extracts^18^. The ABTS radical was generated by mixing equal volumes of ABTS (7 mM) and K_2_S_2_O_8_ (2.45 mM) aqueous solutions and maintaining the mixture in the dark for 16 h. The absorbance of the obtained ABTS ֗^+^ solution was adjusted to 0.7 at 734 nm with ethanol, and different concentrations (25–400 µg/mL) of the investigated extracts were added to the ABTS ֗^+^ solution. The mixture was incubated for 30 min at room temperature in the dark and the absorbance of remaining ABTS ֗^+^ was measured at 734 nm using an automated microplate reader. The antiradical activity of the investigated extracts was compared to that of the standard antioxidant Trolox. Results are presented as % of residual ABTS ֗^+^ radical, with samples containing ABTS radical solution only set as a the control (100%).

IC_50_ values, representing the concentration of extracts/standard antioxidants required to neutralize 50% of DPPH or ABTS֗^+^ radical, were calculated using a nonlinear regression curve fit in GraphPad Prism software for Windows (version 6.01.). The IC_50_ values are presented as mean ± SD from three independent experiments performed in triplicate.

### Antibacterial activity of extracts

For antimicrobial testing, methanol extracts were dissolved in sterile phosphate-buffered saline (PBS) to prepare working solutions. Serial dilutions were made in the corresponding test medium to achieve final extract concentrations of up to 2000 µg/mL. Appropriate controls were included in each assay: (i) a growth control containing bacteria without extract, and (ii) a sterility control containing medium with extract.

Antimicrobial activity was evaluated against Gram-negative pathogens (*Salmonella enterica* subsp. *enterica* serovar Typhimurium (*S*. Typhimurium) ATCC 14028, *Escherichia coli* ATCC 25922, and *Pseudomonas aeruginosa* ATCC 27853), Gram-positive pathogens (*Staphylococcus aureus* ATCC25923 and *Enterococcus faecalis* ATCC29212), and probiotic strains (*Lactobacillus fermentum* BGGO8-54 and *L. plantarum* BGAN8, from the IMGGI Labbank laboratory collection). Pathogens were grown in Luria-Bertani broth (LB), containing 1% (w/v) tryptone, 0.5% (w/v) yeast extract, and 0.5% (w/v) sodium chloride (NaCl), under aerobic conditions, while *Lactobacillus* strains were grown in MRS broth (HiMedia, Thane, India) under strain-specific conditions: *L. plantarum* was incubated aerobically at 30 °C, whereas *L. fermentum* was incubated under microaerophilic conditions (5% CO₂) at 37 °C.

Minimum inhibitory concentration (MIC) and minimum bactericidal concentration (MBC) were determined using a broth microdilution assay in sterile 96-well microplates, using LB broth for pathogenic strains and Hi-Sensitivity™ Test Broth (HiMedia) for *Lactobacillus* strains. The microdilution/iodonitrotetrazolium chloride (INT) viability readout and downstream plating approach for MIC/MBC determination was applied as previously described by Konaté et al.^19^. Overnight cultures were diluted in test medium to yield a final inoculum of approximately 5 × 10⁵ colony-forming units (CFU)/mL per well and mixed with different concentrations of extract. Plates were incubated for 18–24 h (or until clear growth was observed in controls). After incubation, bacterial growth was assessed by adding INT (Sigma-Aldrich, St. Louis, MO, USA) as a viability indicator; metabolically active bacteria reduce INT to a colored formazan product. For both MIC and MBC determination, 100 µL from each well with no visible growth was collected, serially diluted, and spread-plated onto agar plates to enumerate viable bacteria (CFU). The MIC was defined as the lowest extract concentration showing inhibition of growth, while the MBC was defined as the lowest concentration yielding no recoverable colonies upon plating. Antibiotics were tested in parallel as positive controls. Gentamicin (132 µg/mL) was used for pathogenic strains, and erythromycin (10 µg/mL) for probiotics. All assays were performed at least in duplicate in independent biological repeats.

### Immunomodulatory potential of extracts

To examine the immunomodulatory potential of the extracts, cells isolated from the spleens of 10-12-week old male Wistar rats were used. The animals were bred and conventionally housed (12-h light/dark cycle, ambient temperature 22 ± 2 °C, 60% relative humidity, with unlimited access to standard rodent pelleted food and water) at the animal facility of the Institute for Biological Research “Sinisa Stankovic”, Belgrade, Serbia, and kept in group cages before the experiment. In all experiments, animals were anesthetized by intramuscular (i.m.) injection of 0.2 mL/kg body weight (b.w.) of Zoletil 100 (active substances: tiletamine 50 mg/mL, zolazepam 50 mg/mL) purchased from Virbac (Carros, France). After the experiment animals were put under deep anesthesia, deprivation of life included the use of the guillotine.

Animal care and experimental procedures were conducted in accordance with Directive 2010/63/EU on the protection of animals used for experimental and other scientific purposes and were approved by the Veterinary Directorate, Ministry of Agriculture, Forestry and Water Management (No 003325831 2025). The study was also conducted in adherence to the ARRIVE guidelines.

Spleens were aseptically removed from anesthetized animals and mechanically disintegrated by gently teasing the spleen tissue through a 70 μm cell strainer (BD Falcon, BD Bioscience, Bedford, USA). Red blood cells (RBC) were lysed with RBC lysis buffer (0.802 g NH_4_Cl; 0.084 g NaHCO_3_; 0.037 g Na_2_-EDTA; 100 mL H_2_O), and the remaining leukocytes were washed and suspended in complete RPMI-1640 culture medium with 5% fetal bovine serum (FBS, Capricorn Scientific GmbH, Ebsdorfergrund, Germany). Cells were counted using an improved Neubauer hemocytometer. The number of viable cells, determined by trypan blue exclusion assay, exceeded 95%. For all tests, cells (5 × 10^5^ cells/well) were cultivated (at 37 °C in a humidified atmosphere of 5% CO_2_) in a 96-well plate in the presence of *G*. *dinarica* extracts (0, 2, 10, 50, 250 and 1250 µg/mL for toxicity determination, and 0, 2, 10 and 50 µg/mL for other tests) for 48 h. Cell supernatants were obtained by centrifugation (10 min, 800 × g) and used for the measurement of nitric oxide (NO), myeloperoxidase (MPO), and cytokine contents.

Leukotoxicity of extracts was determined using the MTT assay (in which the cells’ mitochondrial dehydrogenases reduce tetrazolium salt to colored end product formazan)^20^. After cultivation of cells in the presence of extracts, MTT (500 µg/mL) was added and incubation continued for another 3 h. The formazan formed was dissolved by overnight incubation with 10% sodium dodecyl sulfate (SDS) − 0.01 N hydrochloric acid (HCl), and the absorbance of the extracted chromogen was read at dual wavelengths (540/670 nm) spectrophotometrically. Non-linear regression was used to determine IC_50_ and IC_20_ values (according to OECD^21^, a substance that causes the death of more than 20% of leukocytes is considered immunotoxic).

For the detection of nitric oxide, the Griess method was employed, which measures the concentration of nitrites, one of the stable products of NO oxidation^22^. Equal volumes (50 µL) of cell supernatant and Griess reagent (0.1% naphtylenediamine dihydrochloride in water and 1% sulfanilamide in 5% phosphoric acid) were incubated for 10 min at room temperature, and absorbance was measured at dual wavelengths (540/670 nm) spectrophotometrically. The concentration of nitrites was determined from a standard curve generated with a known amount of sodium nitrite (NaNO_2_) and expressed as µM.

The assay for determination of MPO activity is based on MPO’s ability to catalyze the oxidation of *o*-dianisidine dihydrochloride in the presence of hydrogen peroxide (H_2_O_2_)^23^. MPO was measured in supernatants of splenocytes by mixing 22.2 µL of supernatant with 11.1 µL of hexadecyltrimethylammonium bromide (HTAB, 0.5% final concentration), followed by the addition of 966.7 µL substrate solution (0.167 mg/mL *o*-dianisidine dihydrochloride and 0.0005% H_2_O_2_ in 50 mmol/L potassium phosphate buffer, pH 6.0). Absorbance was measured spectrophotometrically at 450 nm at three-minute intervals up to ten minutes against the MPO standard.

Concentrations of tumor necrosis factor (TNF), IFN-γ, IL-17, IL-6, and IL-10 were determined using commercially available ELISA sets (R&D Systems, Minneapolis, USA) according to the manufacturer’s instructions. The standard curve generated using known amounts of the recombinant cytokines provided in the respective set was used to calculate cytokine titers.

### Tumoricidal activity

Tumor cell lines used in this study were obtained from ATCC (Rockville, VA, USA), except for 4T1, which was donated by Prof. Nebojša Arsenijević from the Faculty of Medical Sciences, University of Kragujevac, Serbia. Melanoma (B16, B16-F10, A375), breast cancer (4T1, MCF7) and colon cancer cell lines (MC38, HCT116) of human and mouse origin, as well as the healthy human lung fibroblasts (MRC5) cell line, were seeded at appropriate densities in 96-well plates (3 × 10^3^, 2 × 10^3^, 4 × 10^3^, 2 × 10^3^, 6 × 10^3^, 2 × 10^3^, 6 × 10^3^, and 7 × 10^3^, respectively). All cell lines were cultivated in RPMI-1640 (Capricorn Scientific GmbH, Ebsdorfergrund, Germany), except for MC38, which was propagated in DMEM high glucose (Capricorn Scientific GmbH, Ebsdorfergrund, Germany). The medium was supplemented with 0.01% sodium pyruvate, 2 mM L-glutamine, 10% heat-inactivated FBS (Capricorn Scientific GmbH, Ebsdorfergrund, Germany), penicillin (100 units/mL), and streptomycin (100 µg/mL). All cells were grown in T-25 flasks in an incubator at 37 °C in a humidified atmosphere with 5% CO_2_. To determine the potential antitumor activity of extracts obtained from plants collected in natural habitats and grown in vitro, cells were treated with a range of extract doses starting from 200 µg/mL and incubated for 72 h. Stock solutions of all extracts were prepared in DMSO at a concentration of 200 mg/mL, and the starting concentration was 200 µg/mL, for all cell lines. Paclitaxel (Fresenius Kabi, Linz, Austria), derived from the bark of the Pacific yew tree, which served as the positive control was applied starting from a concentration of 50 nM and kept on cells during 72 h. After the incubation period had been ended, cells were washed with PBS and then incubated with MTT solution (AppliChem, St. Louis, MO, USA, 500 µg/mL) at 37 °C under standard conditions for approximately 1 h, depending on the cell lines’ respiration rate. Formed formazan crystals were dissolved in dimethyl sulfoxide (Sigma-Aldrich, St. Louis, MO, USA). Absorbance was measured at 540 nm, with reference at 670 nm, using the SpectraMaxM5 multi-well plate reader (Molecular Devices, San Jose, CA, USA). Cell viability was calculated as a percentage of the control (100%), representing untreated cells. Experiments were performed in triplicate.

### Statistical analysis

The results were obtained from three independent experiments and are presented as mean ± standard deviation. Data were compared using analysis of variance (one-way ANOVA) followed by Tukey’s test (STATISTICA 7.0, StatSoft Inc, Tulsa, OK), and *p*-values < 0.05 were considered significant.

## Results

### Chemical composition of extracts

**Fig. 2:**
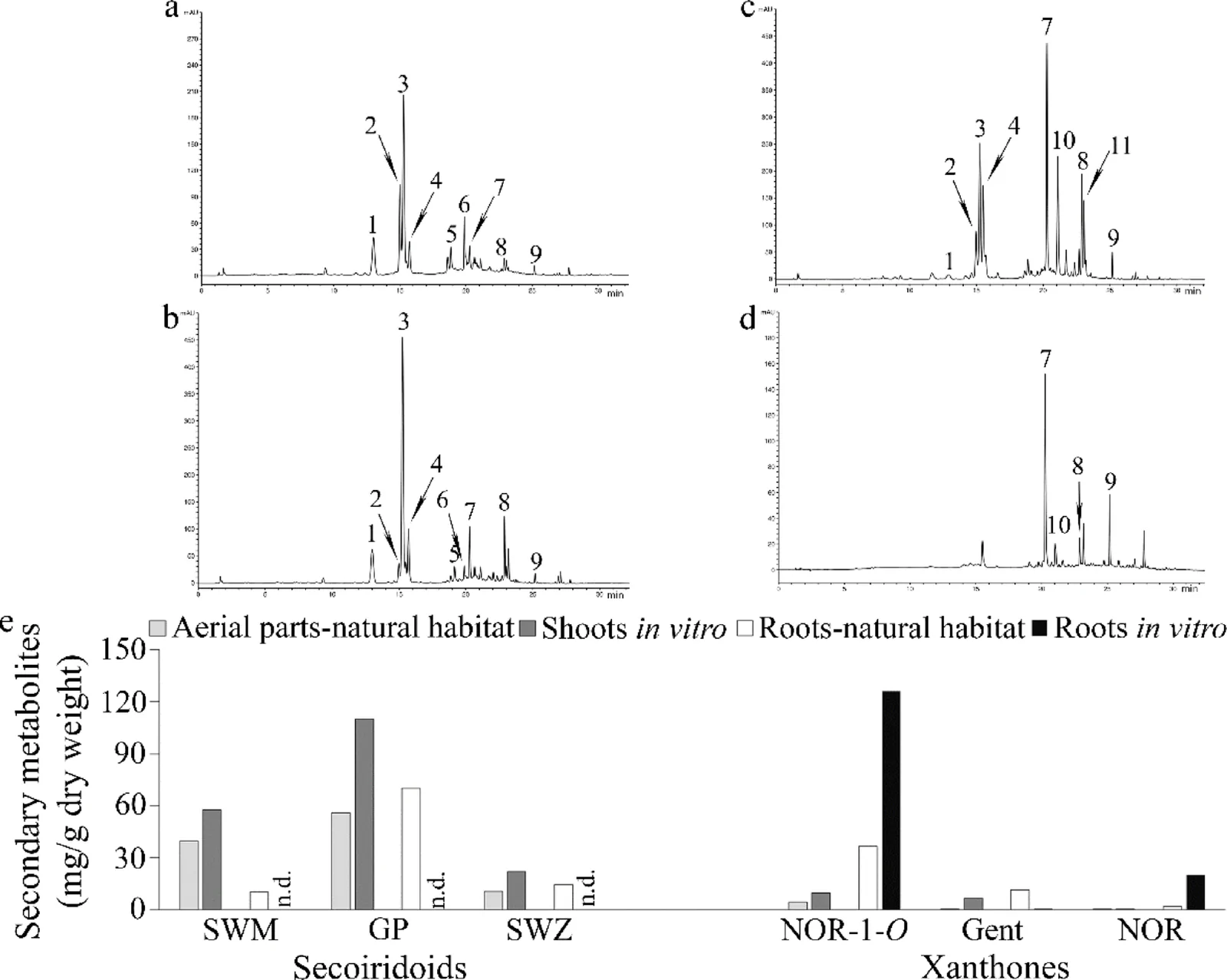
Phytochemical analysis of methanol extracts. Representative HPLC profiles (recorded at 260 nm) of methanol extracts of *G. dinarica*: (**a**) aerial parts from natural habitat, (**b**) shoots cultured in vitro, (**c**) roots from natural habitat, and (**d**) roots cultured in vitro. Peaks − 1- swertiamarin (SWM); 2- isoorientin-4’-*O*-glucoside; 3- gentiopicrin (GP); 4- sweroside (SWZ); 5- isoorientin; 6- isovitexin; 7- norswertianin-1-*O*-primeveroside (NOR-1-*O*); 8- gentioside (Gent); 9- norswertianin (NOR); 10- norswertianin-8-*O*-primeveroside; 11- amarogentin. (**e**) Secondary metabolites content; n.d. – not detected.

As previously reported by Arambašić Jovanović et al.^9^, the total methanol extract of the plant species *G. dinarica* contains secoiridoids and xanthones as the main secondary metabolites, with flavonoids present in smaller amounts. HPLC analysis did not reveal any qualitative differences between extracts from wild plants and those grown in vitro. However, these extracts differ in their quantitative composition (Fig. 2). For example, shoots obtained from in vitro culture contain higher levels of secoiridoids and xanthones than those collected from the natural habitat (*p* < 0.001 for all tested compounds) (Fig. 2a, b, e). The most abundant compounds detected in both aerial parts from the natural habitat and shoot cultures are the secoiridoids gentiopicrin (55.9 mg/g DW and 109.9 mg/g DW, respectively; Fig. 2a, b) and swertiamarin (39.8 mg/g DW and 57.5 mg/g DW, respectively; Fig. 2a, b). The same analysis showed a substantial difference in the chemical composition of root extracts. Specifically, root cultures do not contain secoiridoids, whereas these compounds are present in large quantities in roots from the natural habitats. Conversely, roots cultured in vitro accumulate significant amounts of xanthones (*p* < 0.001 for all tested compounds), especially norswertianin in both glycoside (126.1 mg/g DW) and aglycone forms (19.9 mg/g DW) (Fig. 2c, d, e).

### Antioxidant activity of extracts

**Fig. 3:**
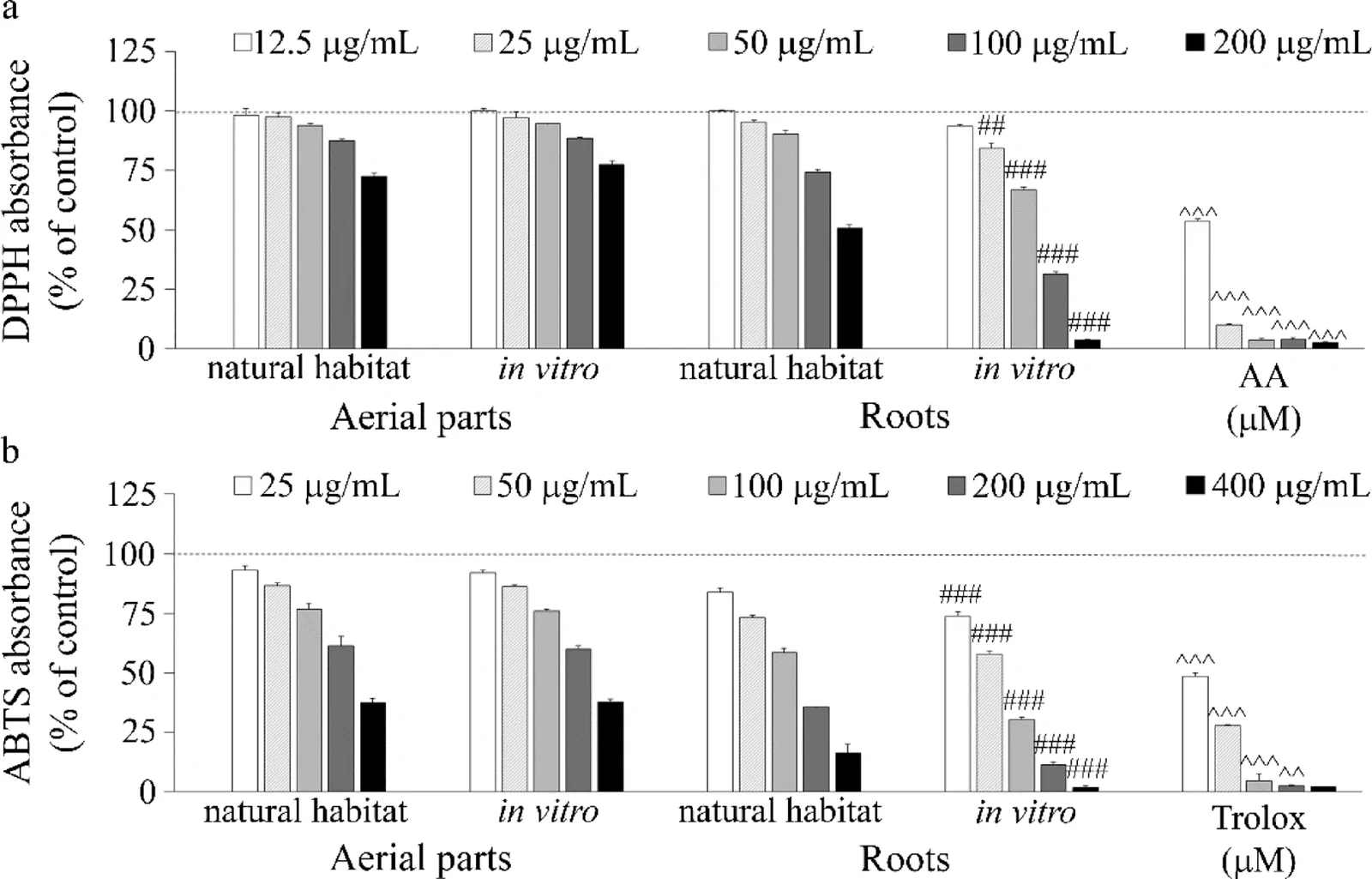
Antioxidant activity of *G. dinarica* extracts. (**a**) DPPH assay. (**b**) ABTS assay. Statistically significant differences between roots cultured in vitro and roots from natural habitat at: ^##^ *p* < 0.01 and ^###^ *p* < 0.001. Results are presented as mean ± SD from three independent experiments performed in triplicate. Statistically significant differences between standard antioxidant (AA – ascorbic acid, Trolox) and roots cultured in vitro at: ^^ *p* < 0.01 and ^^^ *p* < 0.001.

The antioxidant activity of the extracts was evaluated using DPPH (Fig. 3a) and ABTS assays (Fig. 3b). Based on IC_50_ values in the DPPH assay, *G. dinarica* aerial parts and roots grown in the natural habitat, as well as shoot culture, do not exhibit significant antioxidant capacity (Table 1). Similar results were observed in the ABTS assay (Table 1). Roots cultured in vitro showed the highest antioxidant capacity among the examined extracts. The IC_50_ values for the standard antioxidants, ascorbic acid and Trolox, were 12.5 ± 0.4 µM and 23.7 ± 0.4 µM, respectively. Statistical analysis indicated that extracts from roots, both from the natural habitat and cultured in vitro, have greater antioxidant activity than *G. dinarica* aerial parts. Furthermore, the analysis demonstrated a higher capacity of in vitro cultured roots to scavenge DPPH and ABTS radicals compared to roots from the natural habitat.

**Table 1:** Antioxidant activity of *G. dinarica* extracts presented as IC_50_ (μg/mL).

Antioxidant assay	Aerial parts		Roots		Standard antioxidant*
	Natural habitat	In vitro	Natural habitat	In vitro	
*DPPH*	435.7 ± 4.2	458.8 ± 9.9	214.2 ± 16.1	57.6 ± 7.9^###^	12.5 ± 0.4
*ABTS*	269.2 ± 6.8	267.9 ± 3.6	114.0 ± 7.4	55.3 ± 1.6^###^	23.7 ± 0.4

* - standard antioxidant was ascorbic acid for DPPH and Trolox (a water-soluble analog of vitamin E) for ABTS assay; IC_50_ – half maximal inhibitory concentration. The IC_50_ values are presented as mean ± SD from three independent experiments performed in triplicate. Statistically significant differences between roots cultured in vitro and roots from the natural habitat at ### *p* < 0.001.

### Antibacterial activity of extracts

To assess the antibacterial effects of *G. dinarica* extracts, Gram-negative (*S.* Typhimurium, *E. coli*, *P. aeruginosa*) and Gram-positive (*S. aureus*,* E. faecalis*) pathogens, as well as probiotic strains (*L. fermentum* and *L. plantarum*), were grown in the presence of various extract concentrations (up to 2000 µg/mL). The viability and growth of *S.* Typhimurium, *E. coli*, and *E. faecalis* were unaffected by the tested extracts (Table 2). The extract from roots collected in a natural habitat also showed no effect on *P. aeruginosa* growth, whereas other extracts inhibited the growth of this bacterium at different concentrations. Bactericidal activity against *P. aeruginosa* was observed only with the extract from the root cultured in vitro at the highest examined dose. Roots (from both natural habitat and cultured in vitro) and aerial parts from natural habitat displayed bactericidal activity against *S. aureus* at 350 µg/mL, while the extract from shoot cultures eliminated this bacterium at 750 µg/mL. Shoot and root culture extracts inhibited the growth of *L. fermentum* at lower doses than extracts from plant parts collected in natural habitat, with aerial parts from natural habitat showing the lowest antibacterial activity against *L. fermentum*. Bacteriostatic and bactericidal concentrations against *L. plantarum* were similar for the examined extracts.

**Table 2:** Antimicrobial effects of *G. dinarica* extracts presented as MBC / MIC (μg/mL).

Bacterial strains	Aerial parts		Roots		MBC for antibiotic*
	Natural habitat	In vitro	Natural habitat	In vitro	
*Gram negative*					
*S.* Typhimurium	> 2000 / > 2000	> 2000 / > 2000	> 2000 / > 2000	> 2000 / > 2000	132
*E. coli*	> 2000 / > 2000	> 2000 / > 2000	> 2000 / > 2000	> 2000 / > 2000	132
*P. aeruginosa*	> 2000 / 2000	> 2000 / 1500	> 2000 / > 2000	2000 / 1750	132
*Gram positive*					
*S. aureus*	350 / 250	750 / 500	350 / 250	350 / 250	132
*E. faecalis*	> 2000 / > 2000	> 2000 / > 2000	> 2000 / > 2000	> 2000 / > 2000	132
*Probiotics*					
*L. fermentum*	> 2000 / 1500	750 / 500	2000 / 1250	750 / 500	10
*L. plantarum*	1250 / 1000	1250 / 750	1250 / 750	1000 / 750	10

* - antibiotic used as a control for pathogenic strains was gentamicin, and for probiotics was erythromycin; MBC, minimal bactericidal concentration; MIC, minimal inhibitory concentration. Assays were performed at least in duplicate.

### Immunomodulatory potential of extracts

**Fig. 4:**
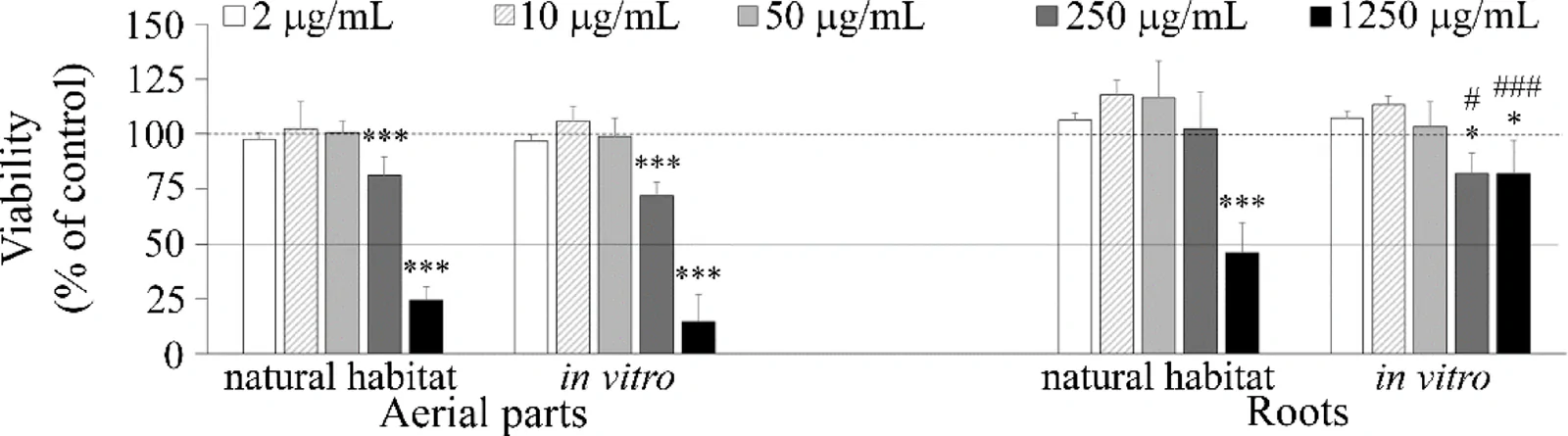
Effect of *G. dinarica* extracts on spleen cell viability. Results are presented as mean ± SD (*n* = 9). Statistically significant compared to control (untreated cells) at: * *p* < 0.05 and *** *p* < 0.001. Statistically significant differences between roots cultured in vitro and roots from natural habitat at: ^#^ *p* < 0.05 and ^###^ *p* < 0.001.

To investigate the immunomodulatory potential of the extracts, spleen cells were cultured with or without *G. dinarica* extracts. Cultivation of spleen cells in the presence of extracts for 48 h showed that plant extracts had similar toxicity, regardless of whether they were obtained from the natural habitat or grown in culture (Fig. 4). Both extracts caused statistically significant toxicity to splenocytes at 250 and 1250 µg/mL, and calculation of the doses causing death of 50% of cells (IC_50_) revealed similar values (819 µg/mL for plants from the natural habitat vs. 714 µg/mL for shoot culture). Extract derived from roots of plants from natural habitat was toxic at 1250 µg/mL (with an IC_50_ value of 1190 µg/mL), while roots cultured in vitro caused approximately 20% toxicity at 250 and 1250 µg/mL. For further functional examinations, only non-toxic doses were selected.

**Fig. 5:**
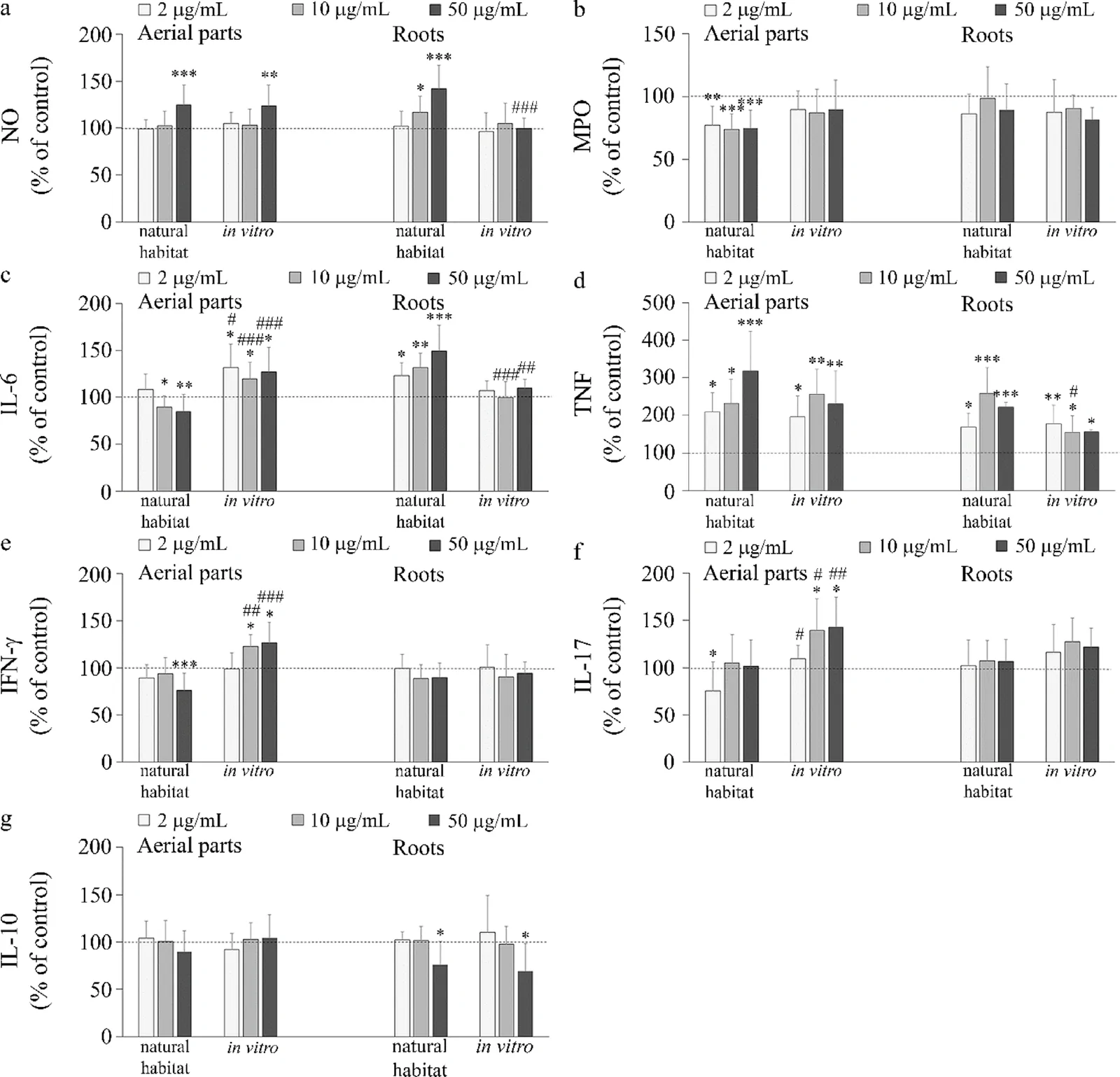
Effects of *G. dinarica* extracts on the production of inflammatory mediators. (**a**) NO, (**b**) MPO, (**c**) IL-6, (**d**) TNF, (**e**) IFN-γ, (**f**) IL-17 and (**g**) IL-10. Results are presented as mean ± SD (*n* = 9). Statistically significant compared to controls (untreated cells) at: * *p* < 0.05, ** *p* < 0.01, and *** *p* < 0.001. Statistically significant differences between plants cultured in vitro and plants obtained from natural habitat at: ^#^ *p* < 0.05, ^##^ *p* < 0.01, and ^###^ *p* < 0.001.

Increased NO production was observed in cells stimulated with both aerial part extracts at 50 µg/mL (with no differences between extracts) and with roots from the natural habitat at 10 and 50 µg/mL, while cultured roots did not affect NO production (Fig. 5a). In contrast, only cells stimulated with aerial part extract from the natural habitat showed a statistically significant decrease in MPO activity at all concentrations (Fig. 5b). Comparison of cytokine production following stimulation with aerial part extracts from the natural habitat or culture revealed an increase in TNF production (Fig. 4d) and no significant changes in IL-10 production (Fig. 5g) in response to both extracts. Unlike plants grown in the natural habitat, which lowered IL-6 (10 and 50 µg/mL), IFN-γ (50 µg/mL) and IL-17 (2 µg/mL), the extract obtained from shoot cultured in vitro caused an increase in the production of these cytokines (IL-6 at 2, 10 and 50 µg/mL; IFN-γ and IL-17 at 10 and 50 µg/mL) (Fig. 5c, e, f). Roots from the natural habitat increased IL-6 and TNF production at all examined doses, decreased the IL-10 response at 50 µg/mL, while no statistically significant changes were noted in IFN-γ and IL-17 production. Extracts derived from roots grown in vitro had similar effects to roots from the natural habitat, except for unchanged IL-6 production and a slightly lower TNF response.

### Tumoricidal activity of extracts

**Fig. 6:**
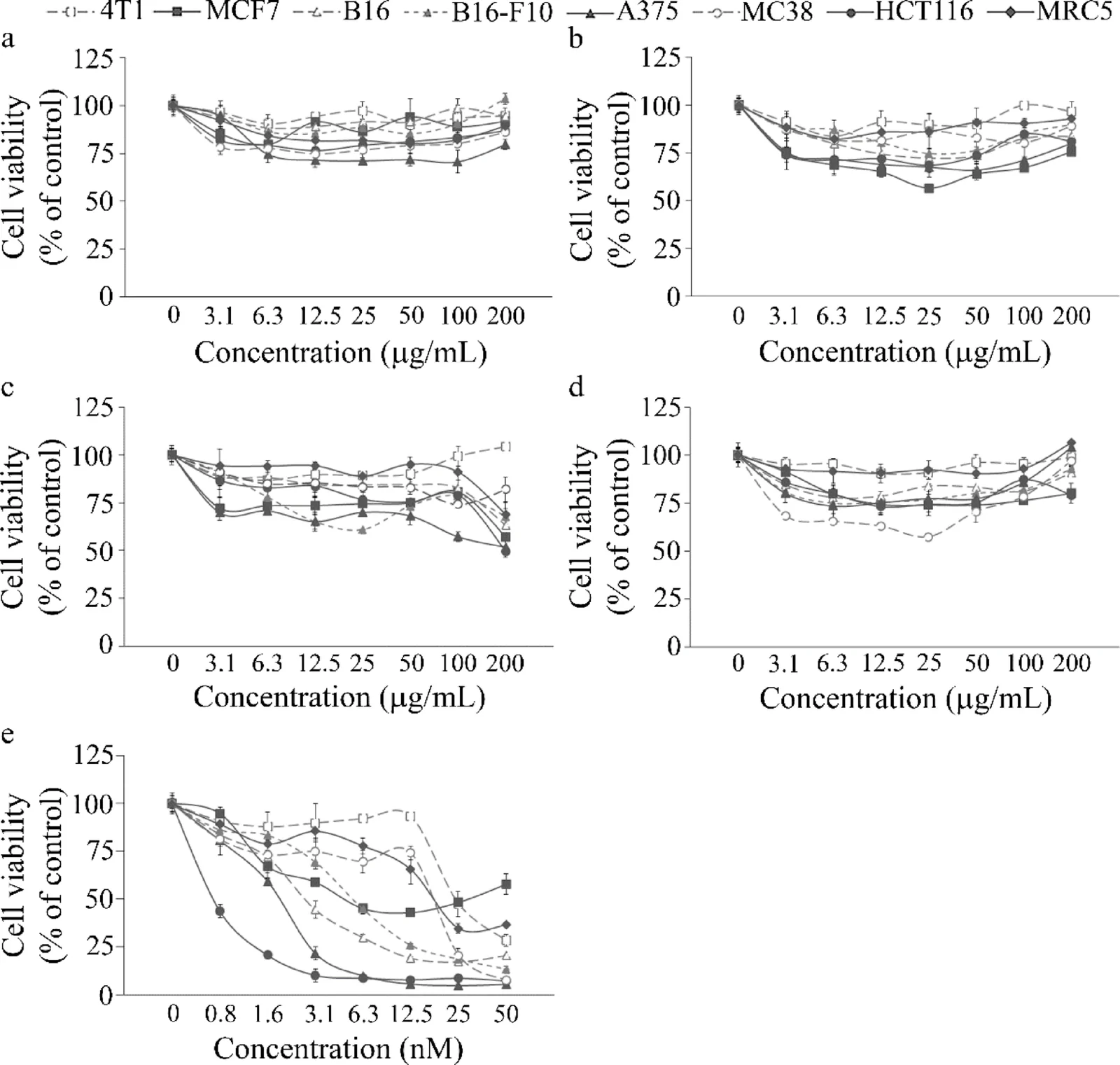
Effect of *G. dinarica* methanol extracts on the viability of tumor cell lines and normal, transformed cell line. (**a**) Aerial parts of plants from natural habitat, (**b**) shoots cultured in vitro, (**c**) roots from natural habitat, (**d**) roots cultured in vitro, and (**e**) paclitaxel.

Selected extracts do not exhibit significant cytotoxic activity against melanoma (B16, B16-F10, A375), breast (4T1, MCF7), or colon (MC38, HCT116) cell lines, as these extracts did not kill up to 50% of the cells even at the highest concentrations tested (except for the extract of roots from natural habitat on HCT116 cells) (Fig. 6). To gain better insight into the effects of different extracts, we analyzed cytotoxicity in tumor cell lines following stimulation with 50 µg/mL of extracts (Table 3). This dose was chosen due to its impact on the immune response, as one of the main components of tumor microenvironment, which may contribute to overall antitumor activity. Extracts derived from aerial parts of plants grown in natural habitat generally did not affect tumor cell viability (except in A375 cells, where a 23.1% decrease in viability was observed). This extract showed no selectivity against tumor cells, as it also caused an 18.1% decrease in the viability of MRC5 cells. Other examined extracts caused less than a 10% decrease in viability in MRC5 cells, and affected tumor cell lines to varying degrees. Shoot culture caused a decrease in viability of 33.3% in MCF7, 28.1% in B16-F10, 32.0% in A375 and 22.4% in HCT116 cells. Extracts derived from both roots affected the viability of selected cell lines to a similar degree, with the best results noted for A375 cells, for which 29.3% (roots from the natural habitat) and 25.1% (roots cultured in vitro) cell death were observed.

**Table 3:** Viability of different cell lines following treatment with 50 μg/mL of *G. dinarica *extracts.

	Breast		Melanoma			Colon		Normal cell line
	4T1	MCF7	B16	B16-F10	A375	MC38	HCT116	MRC5
*Aerial parts*								
Natural habitat	90.7 ± 0.8***	89.7 ± 6.2	87.6 ± 5.5	85.4 ± 0.1	76.9 ± 7.2	79.2 ± 0.2	86.9 ± 8.2	81.9 ± 2.4
In vitro	85.2 ± 6.2	66.7 ± 4.0***^,##^	78.8 ± 7.5*	71.9 ± 6.2**^,#^	68.0 ± 2.8***	81.1 ± 2.6**	77.6 ± 5.8*	92.5 ± 2.1^##^
*Roots*								
Natural habitat	92.2 ± 2.6	79.4 ± 6.4*	79.7 ± 6.7*	77.4 ± 5.6*	70.7 ± 3.5***	88.8 ± 8.3	77.8 ± 3.3**	93.3 ± 2.8
In vitro	93.5 ± 3.7	77.4 ± 4.7*	78.5 ± 7.1*	78.8 ± 1.8**	74.9 ± 3.5**	78.9 ± 7.4*	80.9 ± 7.9*	93.3 ± 4.0

Results are presented as mean ± SD of triplicate. Statistically significant at: * *p* < 0.05, ** *p* < 0.01, and *** *p* < 0.001 for tumor vs. normal cell line. Statistically significant differences between plants cultured in vitro and plants obtained from natural habitats at: ^#^ *p* < 0.05 and ^##^ *p* < 0.01.

## Discussion

Antioxidant, antimicrobial, antitumor, and immunomodulatory activities of plant extracts derived from *G. dinarica* grown in its natural habitat (aerial parts and roots) and propagated in vitro (shoot and root cultures) were compared in this manuscript. The results indicated that some differences exist in the activity of extracts from plants grown in the natural habitat and those cultured in vitro, but a simple correlation between quantitative and/or qualitative phytochemical differences and the activities examined could not be established, probably due to the complexity of interactions among compounds in the crude extracts. Extracts from shoot cultures have higher secoiridoid and xanthone contents, better tumoricidal activity against some tumor cell lines, and better antibacterial activity against *P. aeruginosa* and probiotic bacterial strains, but worse activity against *S. aureus*, than extracts from aerial parts from the natural habitat. Additionally, shoot culture extracts stimulate IL-6, IFN-γ, and IL-17, while aerial part extracts suppress production of these cytokines. Root culture extract exhibits greater antioxidant and bactericidal activity against *P. aeruginosa* and probiotics than roots from the natural habitat, but the same antibacterial activity against *S. aureus*, and similar antitumor and immunomodulatory potential as roots from the natural habitat. It should be noted that root culture extract contains only xanthones, but in higher amounts than extracts from roots from the natural habitat (which also contain secoiridoids).

Similar IC_50_ values obtained for extracts of *G. dinarica* aerial parts grown in the natural habitat and in shoots cultured in vitro in DPPH and ABTS assays indicate no difference in the activity of these two extracts, despite higher secoiridoid and xanthone contents in shoot cultures compared to plants from the natural habitat. Additionally, high IC_50_ values suggest low antioxidant activity for both aerial parts and shoot culture extracts. Root extracts show better antioxidant activity than aerial parts, with cultured roots being better antioxidants than roots from the natural habitat. These results are consistent with previously published data^9^, where higher DPPH scavenging activity was observed for roots cultured in vitro compared to aerial parts and roots of plants from the natural habitat and shoot cultures of *G. dinarica*. Differences in the antioxidant activity of the examined extracts coincided with different xanthone contents, especially with the content of norswertianin and norswertianin-1-*O*-primeveroside^9^. The observed increase in antioxidant activity of roots cultured in vitro could be correlated with a tenfold increase in the content of the tetrahydroxy-oxygenated xanthone norswertianin (NOR). The presence of free hydroxy groups in polyphenolic compounds may confer a strong ability to scavenge free radicals^24^.

Examined *G. dinarica* extracts displayed bactericidal/bacteriostatic activity against *S. aureus*, selected probiotic strains, and *P. aeruginosa*. In general, greater antibacterial activity was observed against Gram-positive than Gram-negative bacteria, which is consistent with previously published data on the antibacterial activity of *Gentiana* species extracts^25–28^. It should also be noted that lower concentrations of extracts were more effective against pathogenic *S. aureus* than against the probiotic strains *L. fermentum* and *L. plantarum*, suggesting preferential activity towards *S. aureus* and supporting the potential of *G. dinarica* extracts as anti-staphylococcal agents. Although previously published data associate higher flavonoid content with better antibacterial activity^27^, no such connection could be established in this study, as extracts differing in total phenol and flavonoid content (data not shown), as well as phytochemical composition, exhibited similar antibacterial activity.

Differences in certain activities towards spleen cells among the extracts are also evident. Extracts from aerial parts grown in the natural habitat exhibited anti-inflammatory activity on splenocytes as previously published^11^, whereas stimulation of cells with shoot culture extracts resulted in increased cytokine production. Some qualitative and quantitative differences were also noted in response to root extracts. Stimulation of cells with extract derived from roots from the natural habitat resulted in an increase in NO and IL-6 production, while roots cultured in vitro did not alter the production of these mediators. In general, the results suggest that extracts of *G. dinarica* containing secoiridoids cause an increase in NO. However, no connection could be established between the levels of secoiridoids and NO, as a similar increase was detected following stimulation with extracts differing in their content of swertiamarin, gentiopicrin, and sweroside. Only extracts of aerial parts from the natural habitat, which have the lowest xanthone, gentiopicrin, and sweroside contents, statistically significantly suppressed the production of some inflammatory mediators. With the increase in secoiridoids and/or xanthones, the cytokine response to extracts changes. It is interesting to note that extracts containing only xanthones do not stimulate IL-6 (in contrast to other extracts) and produce the lowest TNF response among the examined extracts. Additionally, extracts of both roots (with a higher content of xanthones compared to aerial parts and shoot extracts) suppress the IL-10 response to the same extent, despite differences in extract composition. Although some xanthones have been shown to possess anti-inflammatory activity^29^, to our knowledge, there are no data regarding the immunomodulatory potential of norswertianin and gentioside. In contrast, purified sweroside, swertiamarin, and gentiopicrin are known to display anti-inflammatory activity^30^.

The results obtained revealed that *G. dinarica* does not possess significant tumoricidal activity, as 50% killing of tumor cells was not achieved even at the highest concentration. Nevertheless, the overall antitumor potential of *G. dinarica* cannot be ruled out. In this context, semaglutide - despite lacking direct cytotoxic effects on tumor cells in vitro - has been shown to delay tumor onset, growth, and progression in a murine breast cancer model, most likely through the induction of a favorable antitumor immune response^31^. When we analyzed the viability of tumor cells treated with 50 µg/mL, a dose that affects cytokine production relevant for antitumor immunity^32^, a decrease in viability of more than 25% in certain tumor cells, along with moderate selectivity for the malignant phenotype, was observed for extracts of shoot culture and roots from both natural habitat and culture. The results presented in this study suggest that extracts of plants grown in vitro may exert more potent antitumor activity than extracts of plants from natural habitats, due to the induction of a proinflammatory cytokine response, more pronounced tumoricidal activity, and greater selectivity for tumor cells. Both root extracts exhibited comparable antitumor activity across the tested tumor cell lines, with the most pronounced effects observed in human anaplastic melanoma cells. Unexpectedly, more than 25% growth inhibition was recorded in A375 cells, which are considered highly chemoresistant due to their stem-like phenotype. Regarding antitumor activity in relation to phytochemical composition, gentiopicrin^33,34^, sweroside^35,36^, and swertiamarin^37,38^ have been shown to possess antitumor activity, and the better antiglioma effect of various *G. dinarica* root extracts was correlated with higher xanthone content^10^. Our data indicate that root extracts with different compositions have similar activity against the tumor cells examined, except for MC38 cells, where somewhat better results were noted for the extract of roots cultured in vitro.

In conclusion, although biotechnology based on plant tissue culture methods could enable large-scale biomass production, this process alters the phytochemical composition of extracts quantitatively and/or qualitatively and thus might affect their activity. An increase in secoiridoid and, to a lesser extent, xanthone contents in shoots cultured in vitro (compared to extracts from aerial parts of plants from natural habitats) coincided with better antitumor and antibacterial activity. The most pronounced difference in the activity of aerial parts is in the immunomodulatory potential of extracts, where a shift from anti-inflammatory (aerial parts from natural habitats) to proinflammatory (shoots cultured in vitro) activity was observed. Xanthone accumulation accounts for enhanced antioxidant activity of roots cultured in vitro. In general, an alteration in secondary metabolite contents during propagation of plants in vitro may potentiate, suppress, or have no effect on biological activities of extracts (depending on the activity tested), which indicates that a detailed comparison of various biological activities of extracts obtained from plants grown in natural habitats and tissue cultures is needed.

## Data Availability

The datasets used and/or analysed during the current study are available from the corresponding author on reasonable request.

## References

[1] Stevanović, V. & Jakovljević, K. Distribution, ecology, and some taxonomical notes of the genera *Gentiana* L. and *Gentianella* Moench (Gentianaceae) in the Balkans. In: (eds Rybczyński, J. J., Davey, M. R. & Mikuła, A.) The Gentianaceae - volume 1: Characterization and ecology. Berlin, Heidelberg: Springer Berlin Heidelberg; 169–200. 10.1007/978-3-642-54010-3_5. (2014).

[2] Fortini, P., Di Marzio, P., Guarrera, P. M. & Iorizzi, M. Ethnobotanical study on the medicinal plants in the Mainarde Mountains (central-southern Apennine, Italy). *J. Ethnopharmacol.* **184**, 208–218. 10.1016/j.jep.2016.03.010 (2016). 26969402

[3] Idolo, M., Motti, R. & Mazzoleni, S. Ethnobotanical and phytomedicinal knowledge in a long-history protected area, the Abruzzo, Lazio and Molise National Park (Italian Apennines). *J. Ethnopharmacol.* **127**, 379–395. 10.1016/j.jep.2009.10.027 (2010). 19874882

[4] Vinterhalter, B., Krstić-Milošević, D., Janković, T., Zdravković-Korać, S. & Vinterhalter, D. Quantitative determination of secoiridoid and xanthone glycosides of *Gentiana dinarica* Beck cultured *in vitro*. *Acta Physiol. Plant.* **35**, 567–574. 10.1007/s11738-012-1098-4 (2013).

[5] Grzegorczyk, I., Matkowski, A. & Wysokińska, H. Antioxidant activity of extracts from *in vitro* cultures of *Salvia officinalis* L. *Food Chem.* **104**, 536–541. 10.1016/j.foodchem.2006.12.003 (2007).

[6] Khorasani Esmaeili, A., Mat Taha, R., Mohajer, S. & Banisalam, B. Antioxidant activity and total phenolic and flavonoid content of various solvent extracts from *in vivo* and *in vitro* grown *Trifolium pratense* L. (red clover). *Biomed Res Int.* **2015**, 643285 (2015). 10.1155/2015/643285 26064936 PMC4438149

[7] Parsaeimehr, A., Sargsyan, E. & Javidnia, K. A comparative study of the antibacterial, antifungal and antioxidant activity and total content of phenolic compounds of cell cultures and wild plants of three endemic species of *Ephedra*. *Molecules* **15**, 1668–1678. 10.3390/molecules15031668 (2010). 20336006 PMC6257367

[8] Kubica, P. et al. Phenylpropanoid glycoside and phenolic acid profiles and biological activities of biomass extracts from different types of *Verbena officinalis* microshoot cultures and soil-grown plant. *Antioxidants* **11**, 409. 10.3390/antiox11020409 (2022). 35204291 PMC8868826

[9] Arambašić Jovanović, J. et al. Evaluation of the antidiabetic potential of xanthone-rich extracts from *Gentiana dinarica* and *Gentiana utriculosa*. *Int. J. Mol. Sci.* **25**, 9066. 10.3390/ijms25169066 (2024). 39201752 PMC11354890

[10] Tovilovic-Kovacevic, G. et al. Xanthone-rich extract from *Gentiana dinarica* transformed roots and its active component norswertianin induce autophagy and ROS-dependent differentiation of human glioblastoma cell line. *Phytomedicine* **47**, 151–160. 10.1016/j.phymed.2018.03.052 (2018). 30166100

[11] Milijanovic, S. et al. Comparative analysis of *in vitro* immunomodulatory potential of four *Gentiana* species. *Pharm. Biol.* **64**, 435–450. 10.1080/13880209.2026.2639061 (2026). 41795665 PMC12973847

[12] Murashige, T. & Skoog, F. A revised medium for rapid growth and bioassays with tobacco tissue cultures. *Physiol. Plant.* **15**, 473–497 (1962).

[13] Linsmaier, E. M. & Skoog, F. Organic growth factor requirement of tobacco tissue cultures. *Physiol. Plant.* **18**, 100–128 (1965).

[14] Krstić-Milošević, D. et al. Influence of carbohydrate source on xanthone content in root cultures of *Gentiana dinarica* Beck. *Plant. Growth Regul.* **71**, 147–155. 10.1007/s10725-013-9815-6 (2013).

[15] Krstić, D. et al. Phytochemical investigation of *Gentiana dinarica*. *Biochem. Syst. Ecol.* **32**, 937–941. 10.1016/j.bse.2004.03.007 (2004).

[16] Krstić-Milošević, D. Chemical investigation of pharmacologically active secondary metabolites of some *Gentiana* species. Ph.D. thesis. Faculty of Chemistry, University of Belgrade; (2008). http://ibiss-r.rcub.bg.ac.rs/handle/123456789/2737

[17] Atere, T. G., Akinloye, O. A., Ugbaja, R. N., Ojo, D. A. & Dealtry, G. *In vitro* antioxidant capacity and free radical scavenging evaluation of standardized extract of *Costus afer *leaf. *Food Sci. Hum. Well.* **7**, 266–272. 10.1016/j.fshw.2018.09.004 (2018).

[18] Benkhaira, N., Koraichi, S. & Fikri-Benbrahim, K. In *vitro* Methods to Study Antioxidant and Some Biological Activities of Essential Oils: a Review. *Biointerface Res. Appl. Chem.* **3**, 3332–3347. 10.33263/BRIAC123.33323347 (2022).

[19] Konaté, K. et al. Antimicrobial activity of polyphenol-rich fractions from *Sida alba* L. (Malvaceae) against co-trimoxazol-resistant bacteria strains. *Ann. Clin. Microbiol. Antimicrob.* **11**, 5. 10.1186/1476-0711-11-5 (2012). PMC3316130 22364123

[20] Denizot, F. & Lang, R. Rapid colorimetric assay for cell growth and survival: modifications to the tetrazolium dye procedure giving improved sensitivity and reliability. *J. Immunol. Methods*. **89**, 271–277. 10.1016/0022-1759(86)90368-6 (1986). 3486233

[21] OECD. Detailed review paper on *in vitro* test addressing immunotoxicity with a focus on immunosuppression. In: OECD series on testing and assessment, no. 360. Paris: OECD Publishing; 10.1787/667965bc-en. (2022).

[22] Moorcroft, M. J., Davis, J. & Compton, R. G. Detection and determination of nitrate and nitrite: a review. *Talanta* **54**, 785–803. 10.1016/S0039-9140(01)00323-X (2001). 18968301

[23] Orciuolo, E. et al. Effects of *Aspergillus fumigatus* gliotoxin and methylprednisolone on human neutrophils: implications for the pathogenesis of invasive aspergillosis. *J. Leukoc. Biol.* **82**, 839–848. 10.1189/jlb.0207090 (2007). 17626149

[24] Uvarani, C., Chandraprakash, K., Sankaran, M., Ata, A. & Mohan, P. S. Antioxidant and structure–activity relationships of five tetraoxygenated xanthones from *Swertia minor* (Griscb.) Knobl. *Nat. Prod. Res.* **26**, 1265–1270. 10.1080/14786419.2011.561494 (2012). 21985404

[25] Baba, S. A. & Malik, S. A. Evaluation of antioxidant and antibacterial activity of methanolic extracts of *Gentiana kurroo* royle. *Saudi J. Biol. Sci.* **21**, 493–498. 10.1016/j.sjbs.2014.06.004 (2014). 25313286 PMC4191614

[26] Milutinović, M., Dimitrijević-Branković, S. & Rajilić-Stojanović, M. Plant extracts rich in polyphenols as potent modulators in the growth of probiotic and pathogenic intestinal microorganisms. *Front. Nutr.* **8**, 688843. 10.3389/fnut.2021.688843 (2021). 34409062 PMC8366775

[27] Skinder, B. M., Ganai, B. A. & Wani, A. H. Scientific study of *Gentiana kurroo* Royle. *Medicines* **4**, 74. 10.3390/medicines4040074 (2017). 29023411 PMC5750598

[28] Stefanović, O., Ličina, B., Vasić, S., Radojević, I. & Čomić, L. Bioactive extracts of *Gentiana asclepiadea*: antioxidant, antimicrobial, and antibiofilm activity. *Bot. Serb*. **42**, 223–229. 10.5281/zenodo.1468319 (2018).

[29] Feng, L. et al. Immunomodulatory effects of *Lycium barbarum* polysaccharide extract and its uptake behaviors at the cellular level. *Molecules* **25**, 1351. 10.3390/molecules25061351 (2020). 32188121 PMC7145302

[30] Tovilovic-Kovacevic, G., Zogovic, N. & Krstic-Milosevic, D. Secondary metabolites from endangered *Gentiana*, *Gentianella*, *Centaurium*, and *Swertia* species (Gentianaceae): promising natural biotherapeutics. In: (eds Ozturk, M., Egamberdieva, D. & Pešić, M.) Biodiversity and biomedicine. London: Academic; 335–384. 10.1016/B978-0-12-819541-3.00019-0. (2020).

[31] Stanisavljevic, I. et al. Semaglutide decelerates the growth and progression of breast cancer by enhancing the acquired antitumor immunity. *Biomed. Pharmacother*. **181**, 117668. 10.1016/j.biopha.2024.117668 (2024). 39536536

[32] Ramesh, P., Shivde, R., Jaishankar, D., Saleiro, D. & Le Poole, I. C. A palette of cytokines to measure anti-tumor efficacy of T cell-based therapeutics. *Cancers* **13**, 821. 10.3390/cancers13040821 (2021). 33669271 PMC7920025

[33] Chen, Q. et al. Gentiopicroside inhibits the progression of gastric cancer through modulating EGFR/PI3K/AKT signaling pathway. *Eur. J. Med. Res.* **29**, 47. 10.1186/s40001-024-01637-6 (2024). 38212810 PMC10782718

[34] Lu, W., Chu, P., Tang, A., Si, L. & Fang, D. The secoiridoid glycoside gentiopicroside is a USP22 inhibitor with potent antitumor immunotherapeutic activity. *Biomed. Pharmacother*. **177**, 116974. 10.1016/j.biopha.2024.116974 (2024). 38968798 PMC12772646

[35] Han, X-L., Li, J-D., Wang, W-L., Yang, C. & Li, Z-Y. Sweroside eradicated leukemia cells and attenuated pathogenic processes in mice by inducing apoptosis. *Biomed. Pharmacother*. **95**, 477–486. 10.1016/j.biopha.2017.08.007 (2017). 28865368

[36] Huang, S. et al. Anticancer activity of sweroside nanoparticles in prostate cancer bone metastasis in PC-3 cells involved in Wnt/β-catenin signaling pathway. *J. Biomed. Nanotechnol*. **17**, 1960–1971. 10.1166/jbn.2021.3172 (2021). 34706796

[37] Tang, H. et al. Bioinformatics analysis of differentially expressed genes in hepatocellular carcinoma cells exposed to swertiamarin. *J. Cancer*. **10**, 6526–6534. 10.7150/jca.33666 (2019). 31777582 PMC6856900

[38] Xiao, S. et al. Swertiamarin suppresses proliferation, migration, and invasion of hepatocellular carcinoma cells via negative regulation of FRAT1. *Eur. J. Histochem.* **64**, 3169. 10.4081/ejh.2020.3169 (2020). 33131270 PMC7586251

